# LRIG2 regulates cell proliferation, migration and apoptosis of osteosarcoma

**DOI:** 10.1186/s12885-022-10123-3

**Published:** 2022-10-01

**Authors:** Zhi-Qiang Li, Wei-Jie Liao, Bo-Lin Sun, Zhi-Wen Luo, Nan-Shan Zhong, Jia-Bao Wu, Zhi-Li Liu, Jia-Ming Liu

**Affiliations:** 1 Department of Orthopaedic Surgery, the First Affiliated Hospital of Nanchang University, 330006 Nanchang, People’s Republic of China; 2 Institute of Spine and Spinal Cord, Nanchang University, Nanchang, 330006 People’s Republic of China

**Keywords:** Osteosarcoma, LRIG2, proliferation, Migration, Apoptosis

## Abstract

**Background:**

Osteosarcoma (OS) is one of the malignant bone tumors with strong aggressiveness and poor prognosis. Leucine-rich repeats and immunoglobulin-like domains2 (LRIG2) is closely associated with the poor prognosis of a variety of tumors, but the role of LRIG2 in osteosarcoma and the underlying molecular mechanism remains unclear.

**Objective:**

The aim of this study was to determine the function of LRIG2 in OS and the related molecular mechanism on cell proliferation, apoptosis and migration of OS.

**Methods:**

The mRNA and protein expression of LRIG2 in OS tissues and cells was detected by qRT-PCR, western blot (WB) assay and immunohistochemistry (IHC). The cell counting Kit-8 (CCK-8), clone formation, transwell, TdT-mediated dUTP Nick-End Labeling (TUNEL) and WB assay were applied to determine the proliferation, migration and apoptosis abilities of OS cells and its molecular mechanisms. Spontaneous metastasis xenografts were established to confirm the role of LRIG2 in vivo.

**Results:**

LRIG2 exhibited high expression in OS tissues and OS cell lines and the expression of which was significantly correlated with Enneking stage of patients, knockdown LRIG2 expression significantly inhibited OS cell proliferation, migration and enhanced apoptosis. Silencing LRIG2 also suppressed the growth of subcutaneous transplanted tumor in nude mice. Further, the mechanism investigation revealed that the protein level of cell proapoptotic proteins (Bax, caspase9 and caspase3) all increased attributed to LRIG2 deficiency, whereas expression of anti-apoptotic protein BCL2 decreased. LRIG2 silencing led to the decrease phosphorylation of AKT signaling, a decrease expression of vimentin and N-cadherin. Additionally, silencing LRIG2 significantly decreased the rate of tumor growth and tumor size.

**Conclusions:**

LRIG2 acts as an oncogene in osteosarcoma, and it might become a novel target in the treatment of human OS.

**Supplementary Information:**

The online version contains supplementary material available at 10.1186/s12885-022-10123-3.

## Introduction

Osteosarcoma (OS), arised from osteoid tissues, is the most common malignant bone tumor in both young adolescents and children, mainly localizes in the metaphysis of adolescent long bones, and is characterized by invasion and early metastasis [1–3]. Standard treatment for OS mainly is the surgical removal of localized lesions combined with multiple chemotherapeutics including preoperative chemotherapy and postoperative chemotherapy [4]. The 5-year survival rate of patients with non-metastatic diagnosis ranges from 60 to 70%, while which of patients with metastatic OS drops to 20% [5]. Due to its high propensity for distant metastasis, the lack of specificity and sensitivity biomarkers, inter-tumor heterogeneity and drug resistance, the 5-year survival rate for OS patients has not significantly improved during the past decade [6]. Therefore, it is important to investigate the detailed molecular mechanisms of distant metastasis from OS, and to improve the 5-year survival rate of OS patients.

The human leucine-rich repeats and immunoglobulin like domains 2 (LRIG2) is an integral membrane protein belonging to the LRIG protein family [7], which includes three members in humans, LRIG1, LRIG2 and LRIG3, expressed in almost all organs that have been analyzed [7–10]. LRIG genes encode transmembrane glycoproteins were found on the surface of most cells, consists of an extracellular domain (ectodomain), a transmembrane segment and an intracellular portion [11], plays an important role in the maintenance of structural integrity and exerts its function. The gene is localized on chromosome 1p13, consisting of 19 exons, and spans approximately 50 kb, a region frequently deleted in the malignant cell [11]. In recent years, some studies have shown that LRIG gene family was involved in the development processes of multiple tumors, and regulated tumor growth, cell proliferation, invasion and apoptosis [12]. Among them, LRIG2 plays the role as oncogenes or tumor-suppressor genes, which was found to be abnormally expressed in a wide variety of malignancies, such as glioblastomas [13], melanoma [14] and lung cancer [15], etc. However, the expression and functional role of LRIG2 in OS are still unknown.

In this study, we found that LRIG2 was up-regulated in osteosarcoma tissues and cells, and its high expression was associated with Enneking stage and poor prognosis of OS patients. We then explored that silencing LRIG2 inhibited the cell proliferation and cell migration in vitro and in vivo, which can also enhance apoptosis and its mechanism may be by activating the mitochondrial pathway. This study provided the evidence that LRIG2 could be a target for the treatment of OS.

## Materials and methods

### Tissues microarrays

The human OS tissues microarrays (TMA *n* = 50) was purchased from Alena Biotechnology Co., Ltd (Xi'an, China). For immunohistochemistry (IHC) assays, sections were treated by 2-step plus Poly-HRP Anti Rabbit/Mouse IgG Detection Syste(Elabscience, Wuhan, China). Experiments performed according to the instructions, 4 ℃ overnight incubation of anti-LRIG2 and appropriate secondary antibodies were incubated. IHC expression of LRIG2 was determined by two pathologists blinded to the specimens.

### GEPIA2 database analysis

Accessing Gene Expression Profiling Interactive Analysis (GEPIA2) data (http://gepia2.cancerpku.cn/#index), the survival analysis and all 262 sarcoma patients in the database was selected, and the relationship between LRIG2 and the survival prognosis of sarcoma patients was analyzed.

### Cell culture

Human OS cell lines (HOS and 143B) and the normal human osteoblast cell line hFOB1.19 were purchased from the American Type Culture Collection. All cell lines were cultured in the Dulbecco’s Modified Eagle’s Medium (DMEM, Gibco, CA, USA) supplemented with 10% fetal bovine serum (FBS, Gibco, Grand Island, NY.) and maintained in a humidified atmosphere containing 5% CO_2_ at 37 °C.

### Lentivirus-vector construction and cell transfection

Three short hairpin RNAs were designed to silence the expression of LRIG2. The sequences included: shLRIG2-1, 5ʹ-ATCCTGAATGTGGATCTGAAA-3ʹ; shLRIG2-2, 5ʹ- GGGCCTTTGCTGGTGACAGAA-3ʹ; shLRIG2-3, 5ʹ-AGGCAGTCATCAGCAACTTAT-3ʹ. And the results indicated that shLRIG2-2 showed the highest knockdown efficiency. Cells in the exponential phase of growth were seeded in a 6-well plate. 143B and HOS cells were incubated with 1 × 106 lentivirus-transducing units for 24 h (MOI = 100).

### Reverse transcription-quantitative polymerase chain reaction (qRT-PCR)

Total RNA from cells was extracted using TRIzol (Invitrogen; Theo Fisher Scientific, Inc.) according to manufacturer’s protocol. QRT-PCR experiments for mRNA detection were performed with a ChamQTM Universal SYBR® qPCR Master Mix (Vazyme, Nanjing, China) according to the manufacturer’s instructions. The temperature protocol of first-strand cDNA synthesis was: 42 °C for 2 min, 37 °C for 15 min and 85 °C for 5 s. Thermocycler conditions used as follow: 95 °C for 30 s, followed by 40 cycles at 95 °C for 10 s and 60 °C for 30 s. The expression of LRIG2 was normalized to that of GAPDH. Data analysis was performed using the 2 − ΔΔCT method. Detailed information of the primer sequences is shown in Table 1.

**Table 1:** Primer sequences for mRNA detection

Name	Sequence(5’- 3’)
LRIG2-F	TAAACAAGGGGTGGTTGTATGGC
LRIG2-R	CCAATAAGCTCAGACCCACAAAG
GAPDH-F	CCACCCATGGCAAATTCCATGGCA
GAPDH-R	TCTAGACGGCAGGTCAGGTCCACC

*F* Forward primer, *R* Reverse primer

### Western blot (WB) analysis

The proteins were extracted by lysing with RIPA buffer containing 1% protein phosphatase inhibitor. Proteins were separated by SDS-PAGE (10% gel) and the proteins were transferred onto a polyvinylidene fluoride membrane. Membranes were blocked by 5% skimmed milk. All the blots were cut prior to hybridization with antibodies. Subsequently, incubate the primary antibody overnight at 4 °C. Washing the membrane 3 times with 1xTBST, 5 min each time, then add secondary antibody and incubate for 1 h on a shaker at room temperature. Finally, the immunocomplexes were visualized using an enhanced chemiluminescence western blotting kit (Pierce; Thermo Fisher Scientific Inc.).

### Cell proliferation assay

OS cells transfected with sh-LRIG2 or NC were seeded in 96-well plates at 1 × 10^3/well, respectively, and cultured for 24, 48, 72, and 96 h. CCK8 reagents (Dojindo Laboratories, Kumamoto, Japan) were incubated for 3 h and their viability was measured at 450 nm. For colony formation experiments, spread 1000 cells per well in a 6-well plate, fix with 4% paraformaldehyde after 7 days of growth, and stain with crystal violet. More than 50 cells are counted as one colony.

### Cell migration assay

Briefly, 40,000 cells were added to 8 um to the upper chamber, the lower chamber containing 20% of serum added to the medium, and the cells were fixed with 4 percent migration paraformaldehyde after 12 h. The upper chamber erased cell, crystal violet stain for 7 min, and counted using a light microscope.

### Cell apoptosis by TUNEL staining

TUNEL assay for apoptosis was performed by TUNEL Bright Red Apoptosis Detection Kit (Vazyme, Nanjing, China). Cells were cultured for 24 h before using. After washing with PBS, samples were probed with TUNEL detection kit after permeabilization. Cell nuclei were counterstained with DAPI (4',6-diamidino-2-phenylindole), then estimated with Fluorescence microscope.

### Animal experiments

Ten female BALB / c nu / nu mice (6 weeks old, weighing 18 ~ 20 g, Beijing, China) were kept under specific pathogen-free conditions, a temperature of 25 °C, relative humidity of 55 ~ 65%, 12 h light–dark circulation, standard feed and water are free to drink. 2 × 106 HOS cell transfected with shLRIG2 or negative control were inoculated subcutaneously into the flanks of nude mice (two mice per group). These mice were sacrificed after three weeks for orthotopic osteosarcoma spontaneous metastasis animal model, the subcutaneously grown tumors of HOS were harvested and cut into small fragments (2–3 mm), a single fragment was transplanted into the right tibia of anesthetized nude mice and covered with bone wax. These mice (ten mice per group) were closely monitored for tumor growth, and the tumor size was measured every 4 days. The tumor volume is calculated according to the following formula: Tumor volume = 0.5 × width^2^ × length. After 4 weeks, the mice were killed by cervical dislocation, and the tumor weight was measured. Lung metastatic nodules were assessed by H&E staining. The study was approved by the ethics committee of the First Affiliated Hospital of Nanchang University.

### Statistical analysis

Graph Pad Prism 8.0 software was used for statistical analysis. The results are expressed as mean ± standard deviation (SD). All experiments were repeated three times. And *t* test was used to analyze inter-group differences. "NS" for the *P* > 0.05, showing no significant difference; "*" means the *P* < 0.05, showing significant difference; "**" means *P* < 0.01, showing significant difference.

## Results

### LRIG2 is up-regulated in osteosarcoma and relates with poor prognosis

**Fig. 1:**
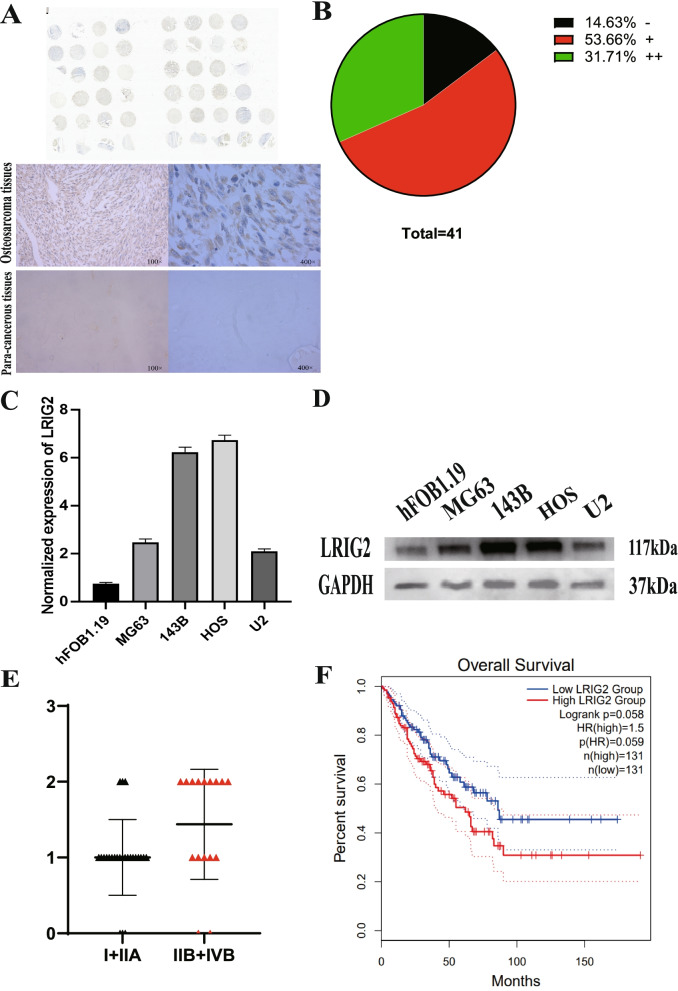
The expressions of LRIG2 in osteosarcoma tissues and cell lines, and its correlation with patients’ prognosis. **A** IHC staining of LRIG2 in OS tissues and Para-cancerous tissues (100 × and 400 ×). **B** Statistical analysis of IHC staining of LRIG2 in human OS TMA. **C** qRT-PCR was performed to measure the expression of LRIG2 mRNA in different OS cells and osteoblastic cells, GAPDH was used as an internal reference. **D** Western blot was performed to measure the relative expression of LRIG2 protein in OS cell lines (MG63, 143B, HOS and U2) compared with human osteoblast cell line hFOB1.19. **E** Distribution of LRIG2 IHC staining scores in OS tissues according to Enneking stage classification. **F** GEPIA2 database analyzes the 5-year survival prognosis of LRIG2 in sarcoma patients

The expression of LRIG2 was examined in OS tissue microarrays through IHC. LRIG2 expression was significantly upregulated in OS tissues compared with that in normal tissues. For 41 patients who were analyzed for tissue expression of LRIG2, positively stained tissues were found in 35 patients (35/41 85.3%), and 53.66% of which had an IHC score of ( +), and 31.71% had an IHC score of (+ +), (Fig. 1A-B). To explore the biological relevance of LRIG2 in OS, the mRNA and protein expression of LRIG2 in indicated OS cells was detected via qRT-PCR and western blot. The results demonstrated that the expression of LRIG2 was significantly increased in MG63, 143B, HOS and U2 cells compared with osteoblasts (Fig. 1C-D).

Together with the clinical data derived from the patient OS tissues, LRIG2 expression was significantly associated with the Enneking stage of OS patients (Fig. 1E). Kaplan–Meier survival curves of OS based on LRIG2 expression using the online bioinformatics tool GEPIA2, and the results indicated that there was a correlation between the expression of LRIG2 and the overall survival of sarcoma patients (Fig. 1F).

### Silencing LRIG2 decreased the proliferation, migration and promoted apoptosis of OS cells

**Fig. 2:**
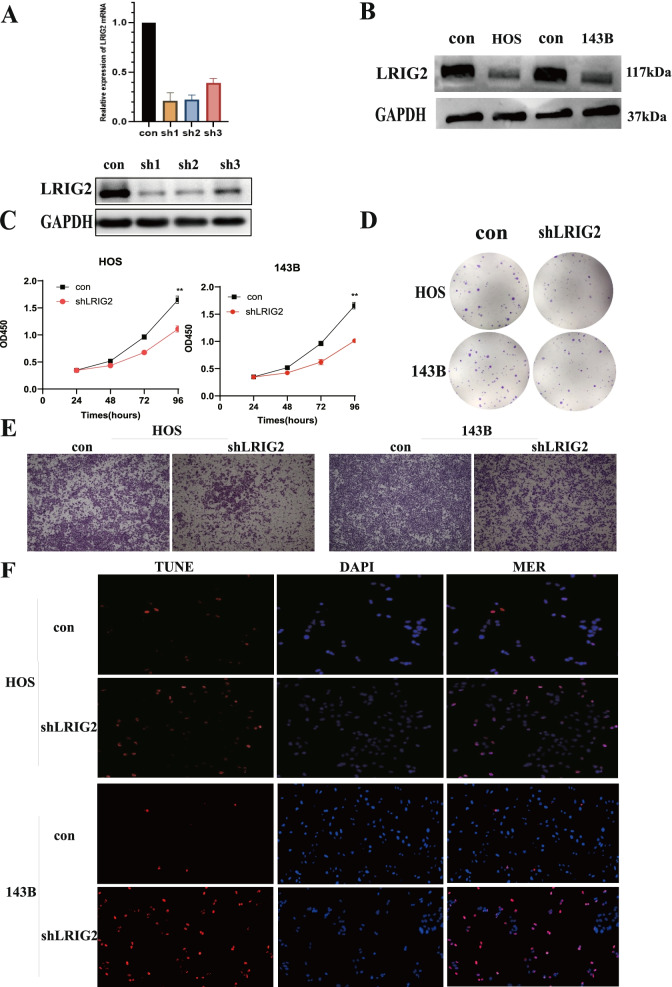
Silencing LRIG2 decreased the proliferation, migration and promoted the apoptosis of osteosarcoma cells. **A **qRT-PCR and western blot analysis the knockout efficiency of LRIG2 gene of three different short hairpins. **B** Western blot analysis for silencing LRIG2 efficiency (HOS and 143B cells). **C** and **D** CCK-8 assay and colony formation was performed to detect the cell proliferation ability. **E** Transwell assay was performed to determine the migration ability of HOS and 143B cells. Representative images showed migrative cells in the lower chamber stained with crystal violet. **F** TUNEL assay was used to detect the apoptosis of osteosarcoma cells. Values presented as mean ± SD. *P < 0.05, **P < 0.01

To explore the role of LRIG2 on OS malignant phenotypes, endogenous LRIG2 expression was silenced in OS cells by using shRNA technology (Fig. 2A-B). To determine the effects of LRIG2 on OS cell viability, we used CCK-8 assay and cloning formation assays. The results showed that silencing LRIG2 significantly decreased the proliferation and colony numbers of OS cells (Fig. 2C-D). These findings indicated that silencing LRIG2 suppressed the proliferation of OS cells. Transwell migration assay was also used to validate the effect of LRIG2 on OS cells migration (Fig. 2E). The results indicated that silencing LRIG2 significantly reduced the migration ability of OS cells. A TUNEL assay was performed to investigate the role of LRIG2 on apoptosis level of OS cells, and the results showed silencing LRIG2 significantly promoted OS cells apoptosis (Fig. 2F).

### The potential molecular mechanism of acquiring a malignant phenotype of OS cells by LRIG2

**Fig. 3:**
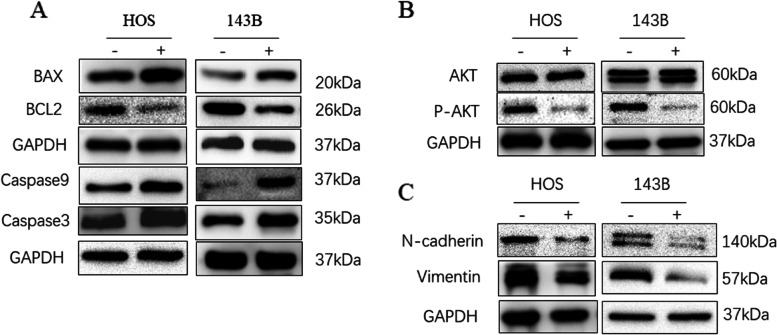
The potential molecular mechanism of acquiring a malignant phenotype of osteosarcoma cells by silencing LRIG2. All the blots were cut prior to hybridization with antibodies. **A** Western blot was used to analyze the apoptosis related protein (BAX, BCL2, caspase9 and caspase3) in OS cells transfected with sh-LRIG2 or NC. **B** Western blots of AKT, p-AKT in OS cells. **C **Western blots of N-cadherin, Vimentin in OS cells

To make clear the potential molecular mechanisms of LRIG2 impacting the malignancy behaviors of OS cells, relative protein levels were measured by western blotting. Western blot assay displayed that the protein levels of cell proapoptotic proteins (Bax, caspase9 and caspase3) all increased attributing to LRIG2 deficiency, whereas the expression of anti-apoptotic protein BCL2 decreased (Fig. 3A). Silencing LRIG2 resulted in the decrease of phosphorylation of AKT signaling, but here was no significant change in the total protein level of AKT (Fig. 3B). In addition, a decreased expression of vimentin and N-cadherin were noted while LRIG2 was silenced (Fig. 3C).

### Silencing LRIG2 inhibits the growth of OS xenograft tumor

**Fig. 4:**
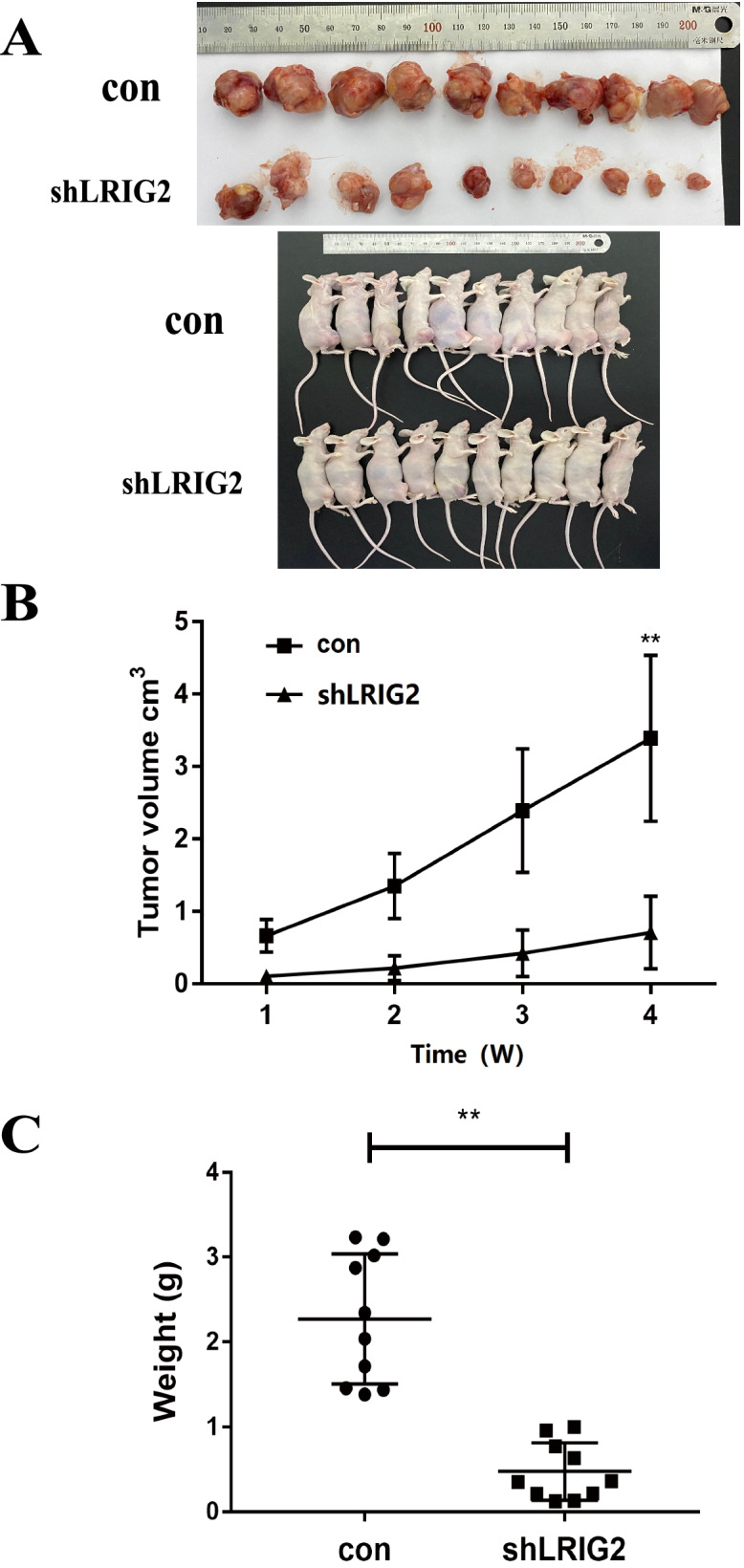
Silencing LRIG2 inhibits the growth of osteosarcoma spontaneous metastasis xenograft models. **A** Orthotopic osteosarcoma xenograft tumor models were established, the nude mice were euthanized after 4 weeks, orthotopic tumors dissected to obtain samples. **B** Tumor sizes were measured weekly and calculated using the following formula: V = (Length × Width^2/2). **C** Tumors were dissected and weighted

To implore the effect of LRIG2 on the tumorigenic potential in vivo, LRIG2 deficiency HOS cell and NC were subcutaneously injected into the flanks of nude mice. We found that silencing LRIG2 significantly decreased the rate of tumor growth and tumor size. What’s more, the orthotopic osteosarcoma spontaneous metastasis animal model showed the same results. These findings indicated silencing LRIG2 could inhibit tumor formation and growth in cell experimental subcutaneous xenograft tumor model in nude mice (Fig. 4A-C).

## Discussion

In the present study, we reported that LRIG2 expression was significantly higher in OS tissues or cells compared with human osteoblast cells, and positively correlated with the Enneking stage of OS patients. These results indicated that LRIG2 might serve as a negative prognostic factor associated with poor OS survival. Similar results were also reported in several other tumors, such as glioma [13], small cell lung cancers [15], skin cancer [16], oligodendrogliomas [17] and early-stage squamous cell carcinoma of the uterine cervix [18], in which LRIG2 was reported up-regulating in such tumor tissues and negatively correlated with the prognosis of patients.

Up to now, the role of gene LRIG2 in OS is unclear. According to the studies of Wang B et al. and Xiao Q et al., downregulation of LRIG2 inhibited glioblastoma cell growth and increased spontaneous apoptosis in vitro [13, 19]. In order to make clear the role of LRIG2 in OS and explore the possible underlying mechanisms, we established OS cells with low expressions of LRIG2 by using shRNA technology. We found that downregulation of LRIG2 inhibited the proliferation of OS cells in vitro and in vivo. Additionally, it also inhibited the cell migration and enhanced the apoptosis of OS cells.

Previous studies revealed that LRIG2 was abnormally over-expressed in a number of tumors and promoted tumor progression. Here, GEPIA2 database was used to evaluate the prognostic value of LRIG2 in sarcoma and the *P* value for the statistics was 0.059. The reason of this may be that the sample contained other types of sarcomas. However, according to the trend of the curve and the analysis of clinical specimen data, there was a certain positive correlation between LRIG2 expression and poor survival prognosis of OS patients.

Studies have shown that LRIG2 participated in the initiation and development of glioma, and promoted cell proliferation through activating the tyrosine kinase and its downstream pathway, also inhibited the spontaneous apoptosis of glioblastoma cells through mitochondrial pathway, and collectively involving in drug resistance [13, 20, 21]. Moreover, LRIG2 exerted a proangiogenic effect by stimulating VEGF production in glioma [21]. In endometrial cancer, LRIG2 promotes the expression of anti-apoptotic proteins and inhibits the expression of pro-apoptotic protein [22]. This ultimately leads to the inhibition of apoptosis of cancerous cells. In the present study, we for the first time demonstrated that silencing LRIG2 could decrease the protein levels of AKT phosphorylation and inhibit the proliferation of OS cells, and downregulation of LRIG2 also inhibited OS tumor formation and growth in cell experimental subcutaneous xenograft tumor model in nude mice. These results were similar to the study of Xiao Q et al., in which the authors indicated that LRIG2 could enhance the activation of EGFR and its downstream PI3K/Akt pathway [20]. In addition, our results revealed that silencing LRIG2 inhibited the cell migration ability by decreasing the expression of vimentin and N-cadherin. These results were not consistent with the study of Wang B et al., in which it demonstrated that knockdown of LRIG2 could increase the adhesive and invasive capability of glioblastoma cell [19]. Thus, LRIG2 may have a completely different role in different types of tumors. In vivo, there was no significant difference for lung metastases in either group. The reason for this may be the observation time was not long enough.

Apoptosis, also called programmed cell death, is a mechanism for the removal of unwanted and damaged cells in the maintenance of normal tissue homeostasis [23–25]. The resistance to apoptosis will cause tumorigenesis and results in cancer cells resistance to chemotherapy and radiotherapy [26–29]. In line with previous studies, our study showed that silencing LRIG2 enhanced the apoptosis of OS cell, due to the pro-apoptotic proteins increased and anti-apoptotic proteins decreased. It may suppress the apoptosis of OS cells through the mitochondrial pathway [30]. These results were consistent with those studies of Wang B et al. and Xiao Q et al. [19, 20].

LRIG1 is another member of LRIG protein family. It was reported that the expression of LRIG1 was negatively correlated with tumor grade and associated with better survival in numerous tumors [31–33], which indicated that the functions of LRIG2 and LRIG1 were different in the progression of tumors. Although it had been reported that the amino acid sequence of LRIG1 was 47% identical to the sequences of LRIG2 [7, 9], the mechanism underlying the different functions between LRIG2 and LRIG1 was still insufficient and required further exploration. In previous study, Mao F et al. and Ye F et al. demonstrated that LRIG1 inhibited the proliferation and promoted the apoptosis of glioblastoma cells in vitro and in vivo [34, 35]. These results suggested that LRIG1 exerted a function as a tumor suppressor, which was in contrast with the function of LRIG2 herein presented.

Taken together, we were the first one to report that LRIG2 expression levels positively associated with Enneking stage, and silencing LRIG2 could inhibit the cell proliferation in vitro and in vivo, inhibit the cell migration of OS and enhance the apoptosis of OS cells. These data in this study provide an experimental basis for further understanding the biological mechanism of LRIG gene family in OS and more experiments are needed to elucidate the mechanism about the functions of LRIG2 in OS cells. It would be important to determine how LRIG2 functioned in OS cells and other tumor cells. We believe that LRIG2 may be a potential therapeutic target for OS treatment in the future.

## Supplementary Information

**Additional file 1:** **Figure 1.** (A) IHC staining of LRIG2 in OS tissues and Para-cancerous tissues (100× and 400×). (B) The images of the original blots of the relative expression of LRIG2 protein in OS cell lines (MG63, 143B, HOS and U2) compared with human osteoblast cell line hFOB1.19. (C) TCGA database analyzes the 5-year survival prognosis of LRIG2 in sarcoma patients. **Figure 2.** (A) The original western blot images of knockout efficiency of LRIG2 gene of three different short hairpins. (B) The original blots images of silencing LRIG2 efficiency (HOS and 143B cells). **Figure 3.** (A) Original western blot images of the expression levels of various proteins after LRIG2 knockdown (HOS cell line). (B) Original western blot images of the expression levels of various proteins after LRIG2 knockdown (143B cell line). All the blots were cut prior to hybridization with antibodies. **Figure 4.** Silencing LRIG2 inhibits the growth of osteosarcoma spontaneous metastasis xenograft models. (A) Orthotopic osteosarcoma xenograft tumor models were established, the nude mice were euthanized after 4 weeks, orthotopic tumors dissected to obtain samples. (B) Tumor sizes were measured weekly and calculated using the following formula: V= (Length×Width^2/2). (C) Tumors were dissected and weighted. **Figure 5.** Silencing LRIG2 inhibits the growth of osteosarcoma xenograft tumor. LRIG2 stably transfected gene silencing were inoculated subcutaneously into the nude mice. The nude mice were euthanized after 5 weeks, subcutaneous tumors dissected to obtain samples (6 for control group and 6 for LRIG2- knockdown group). The relative mRNA expression levels of control group and LRIG2-knockdown group were detected by qRT-PCR.

## Data Availability

All data generated or analyzed during this study are included in this article.
