# Supplementary material for: LRIG2 regulates cell proliferation, migration and apoptosis of osteosarcoma
Source: BMC Cancer. 2022 Oct 1;22:1029. doi: 10.1186/s12885-022-10123-3 (PMC9526349; doi:10.1186/s12885-022-10123-3)
Supplement: Supplementary file 1 — Additional file 1: Figure 1. (A) IHC staining of LRIG2 in OS tissues and Para-cancerous tissues (100× and 400×). (B) The images of the original blots of the relative expression of LRIG2 protein in OS cell lines (MG63, 143B, HOS and U2) compared with human osteoblast cell line hFOB1.19. (C) TCGA database analyzes the 5-year survival prognosis of LRIG2 in sarcoma patients. Figure 2. (A) The original western blot images of knockout efficiency of LRIG2 gene of three different short hairpins. (B) The original blots images of silencing LRIG2 efficiency (HOS and 143B cells). Figure 3. (A) Original western blot images of the expression levels of various proteins after LRIG2 knockdown (HOS cell line). (B) Original western blot images of the expression levels of various proteins after LRIG2 knockdown (143B cell line). All the blots were cut prior to hybridization with antibodies. Figure 4. Silencing LRIG2 inhibits the growth of osteosarcoma spontaneous metastasis xenograft models. (A) Orthotopic osteosarcoma xenograft tumor models were established, the nude mice were euthanized after 4 weeks, orthotopic tumors dissected to obtain samples. (B) Tumor sizes were measured weekly and calculated using the following formula: V= (Length×Width^2/2). (C) Tumors were dissected and weighted. Figure 5. Silencing LRIG2 inhibits the growth of osteosarcoma xenograft tumor. LRIG2 stably transfected gene silencing were inoculated subcutaneously into the nude mice. The nude mice were euthanized after 5 weeks, subcutaneous tumors dissected to obtain samples (6 for control group and 6 for LRIG2- knockdown group). The relative mRNA expression levels of control group and LRIG2-knockdown group were detected by qRT-PCR. [file 12885_2022_10123_MOESM1_ESM.docx]

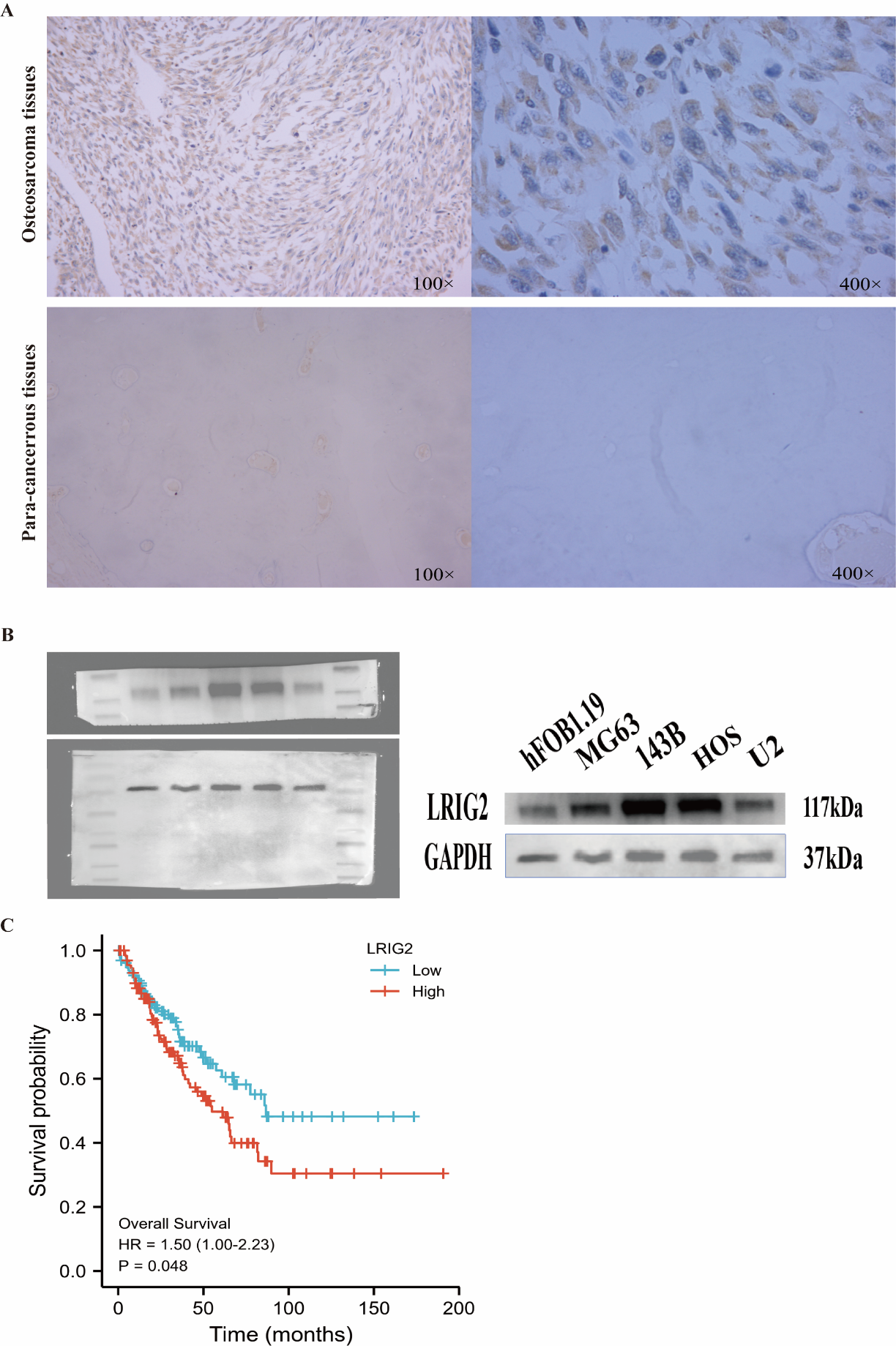


**Figure1.** (A) IHC staining of LRIG2 in OS tissues and Para-cancerous tissues (100× and 400×). (B) The images of the original blots of the relative expression of LRIG2 protein in OS cell lines (MG63, 143B, HOS and U2) compared with human osteoblast cell line hFOB1.19. (C) TCGA database analyzes the 5-year survival prognosis of LRIG2 in sarcoma patients.


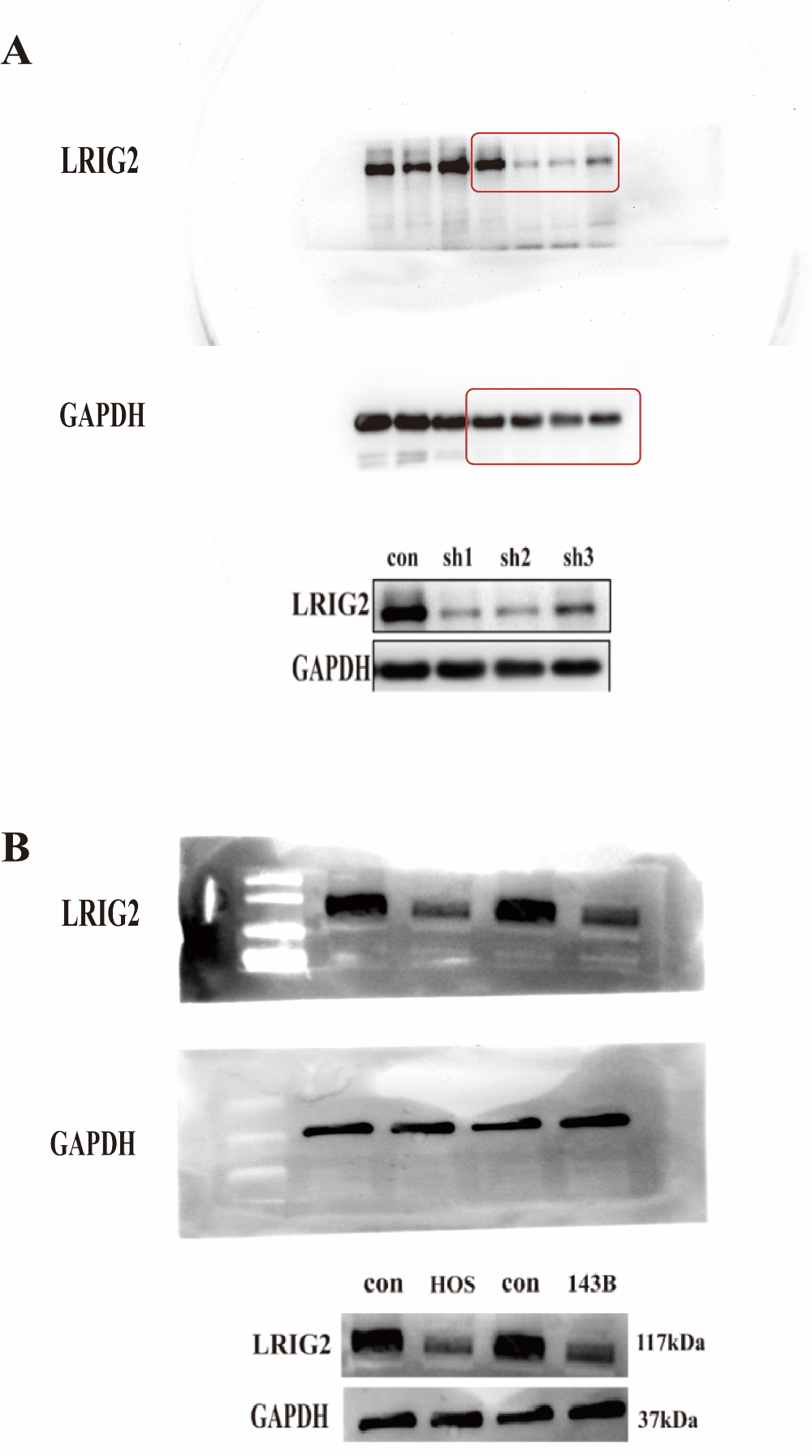


**Figure2.** (A) The original western blot images of knockout efficiency of LRIG2 gene of three different short hairpins. (B) The original blots images of silencing LRIG2 efficiency (HOS and 143B cells).
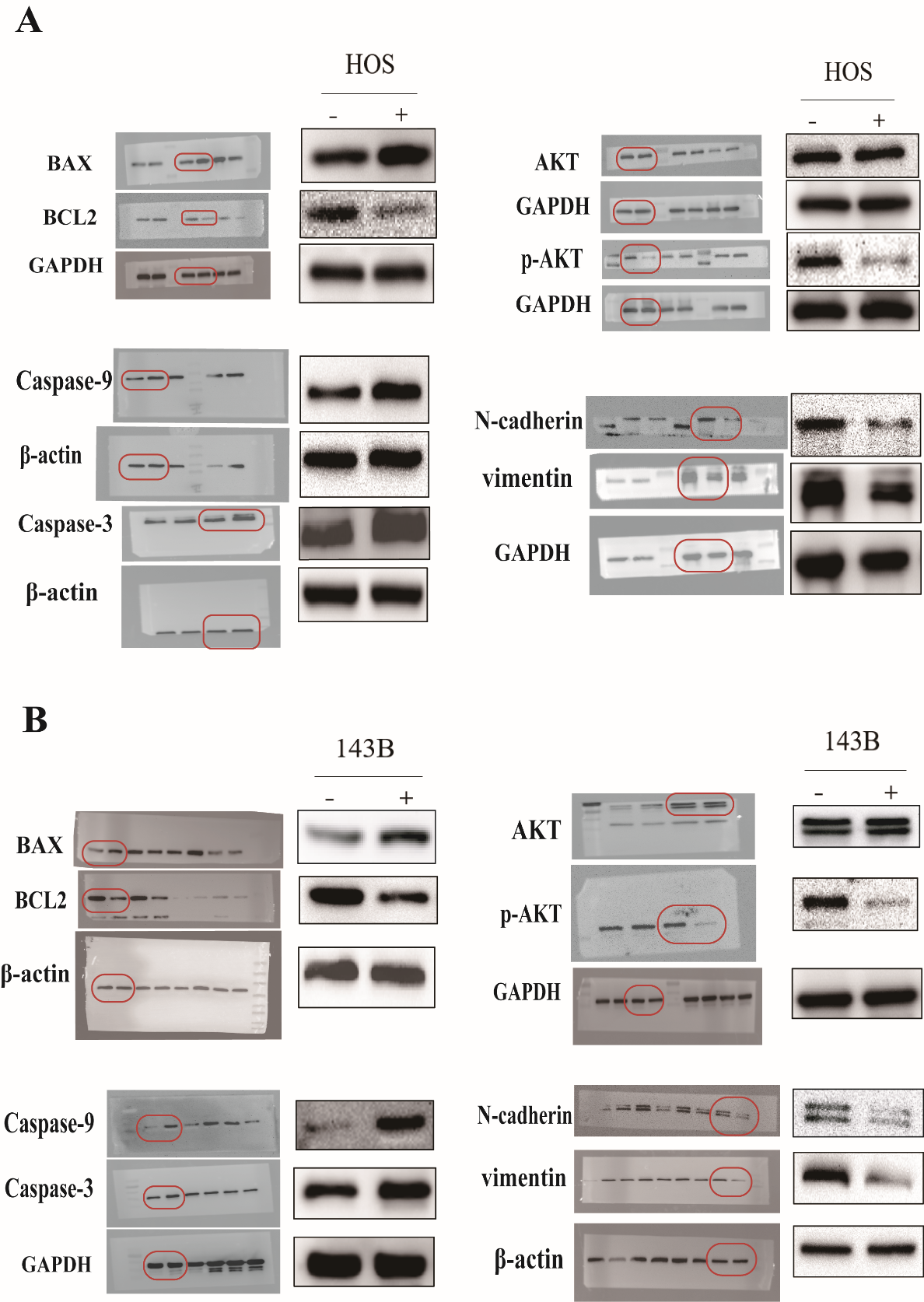


**Figure 3.** (A) Original western blot images of the expression levels of various proteins after LRIG2 knockdown (HOS cell line). (B) Original western blot images of the expression levels of various proteins after LRIG2 knockdown (143B cell line). All the blots were cut prior to hybridization with antibodies.


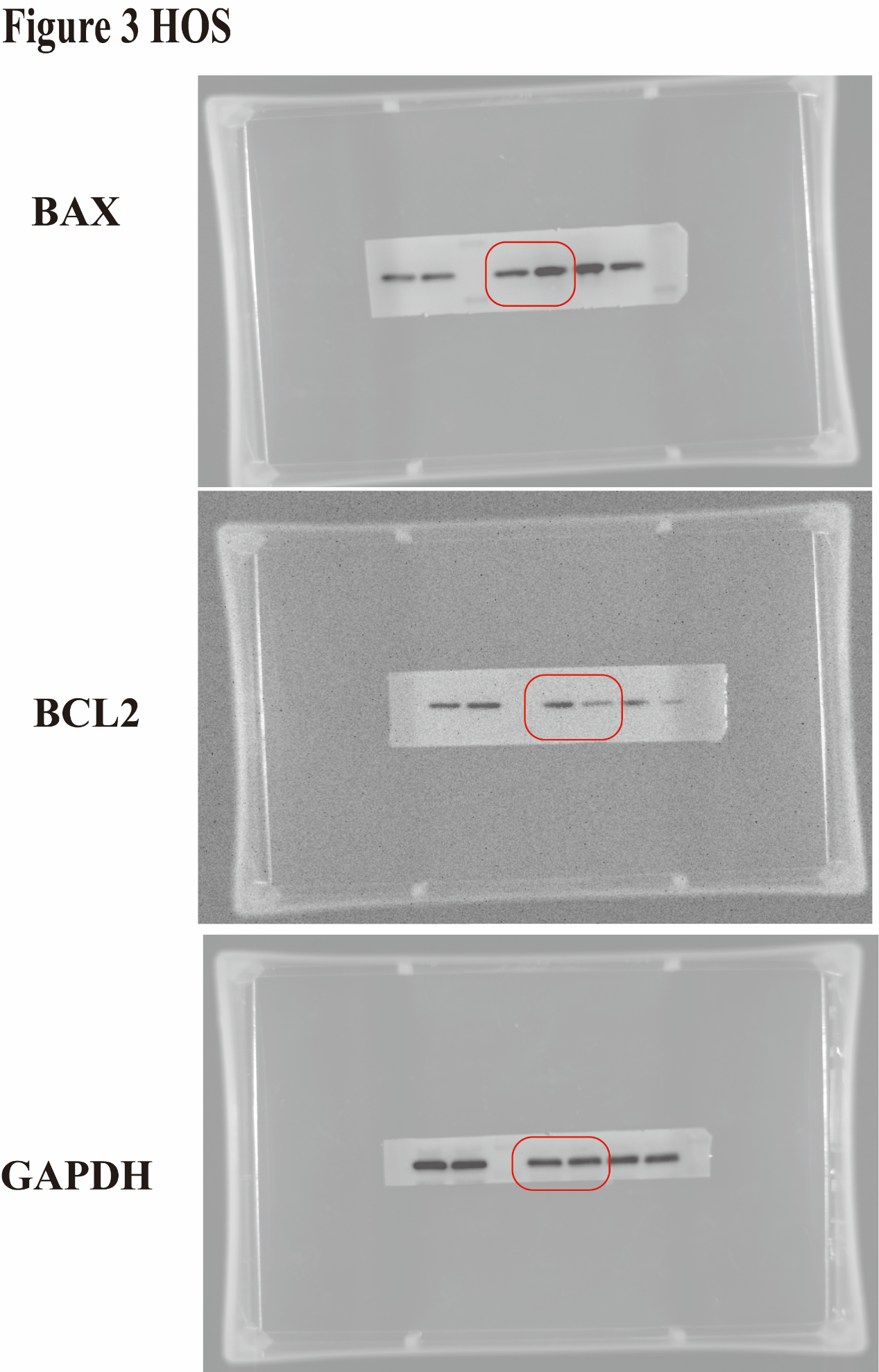


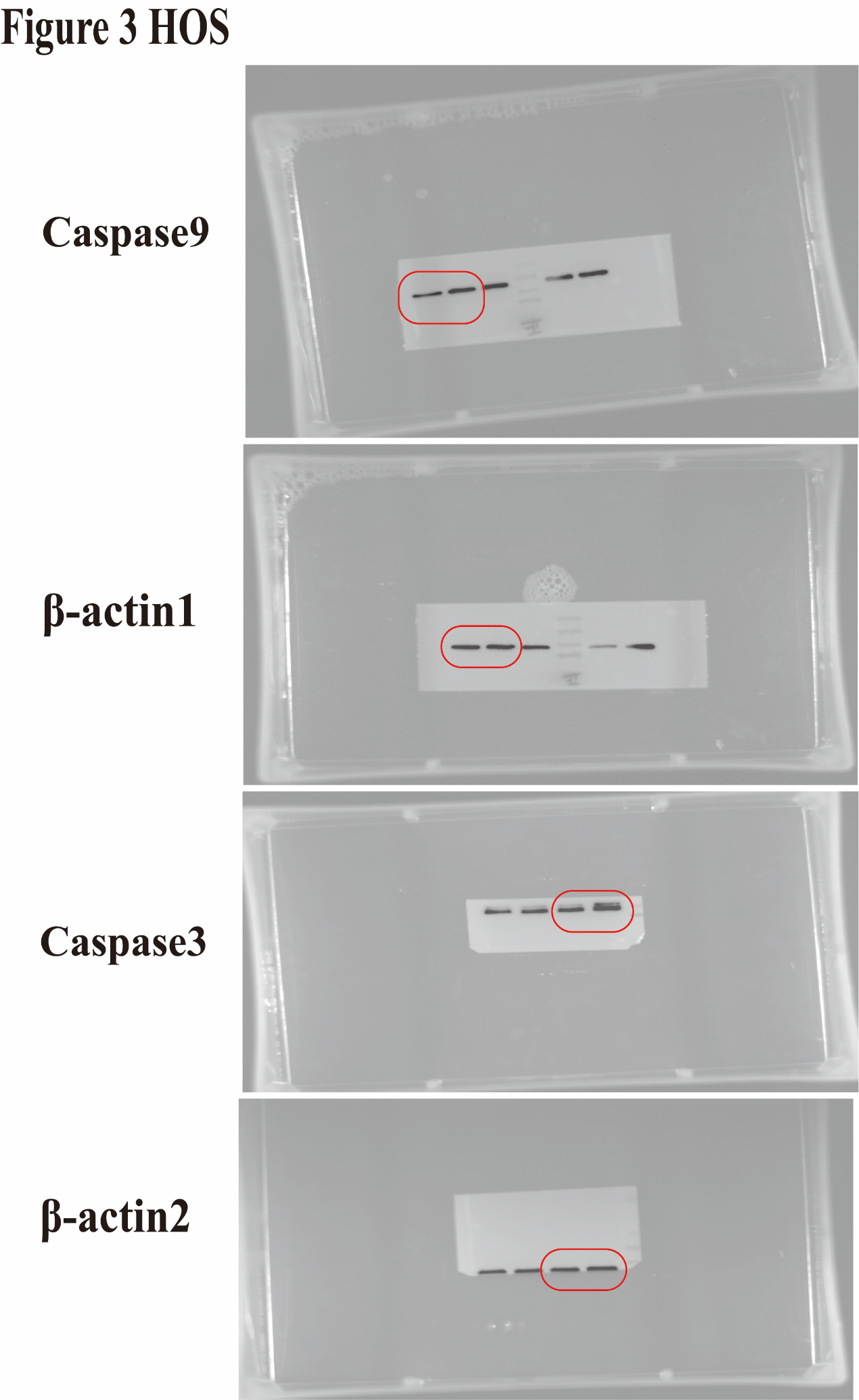


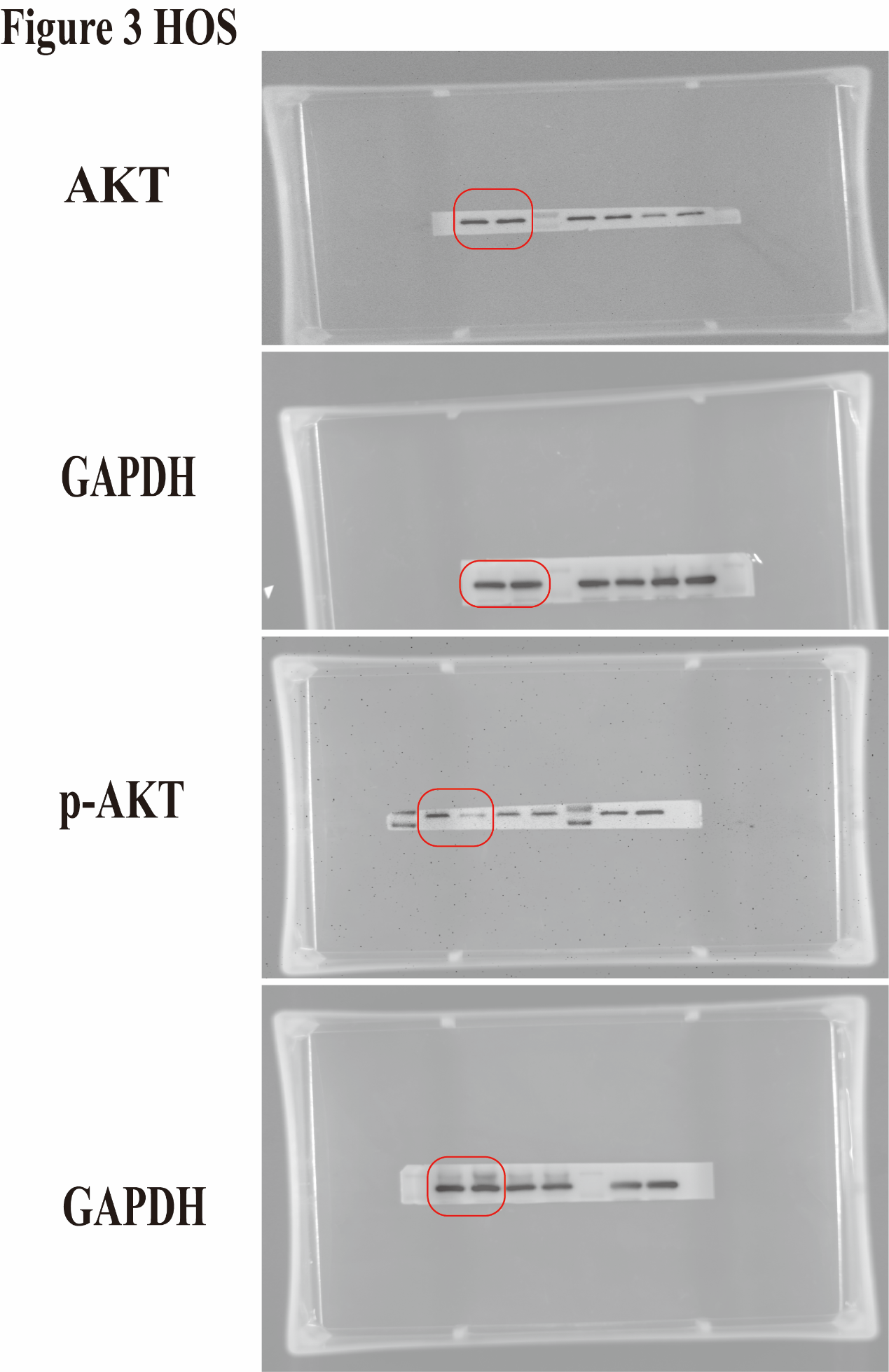


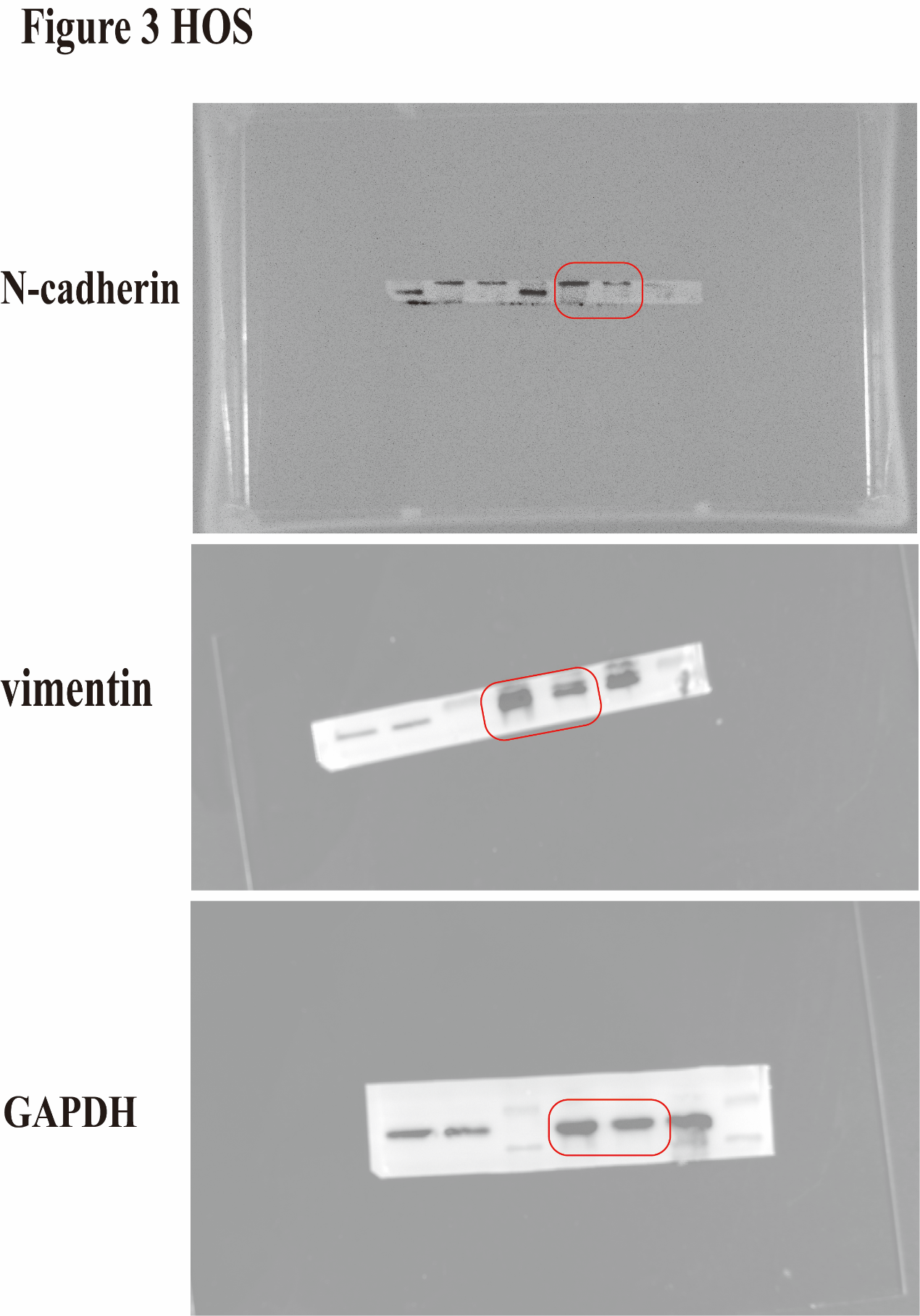


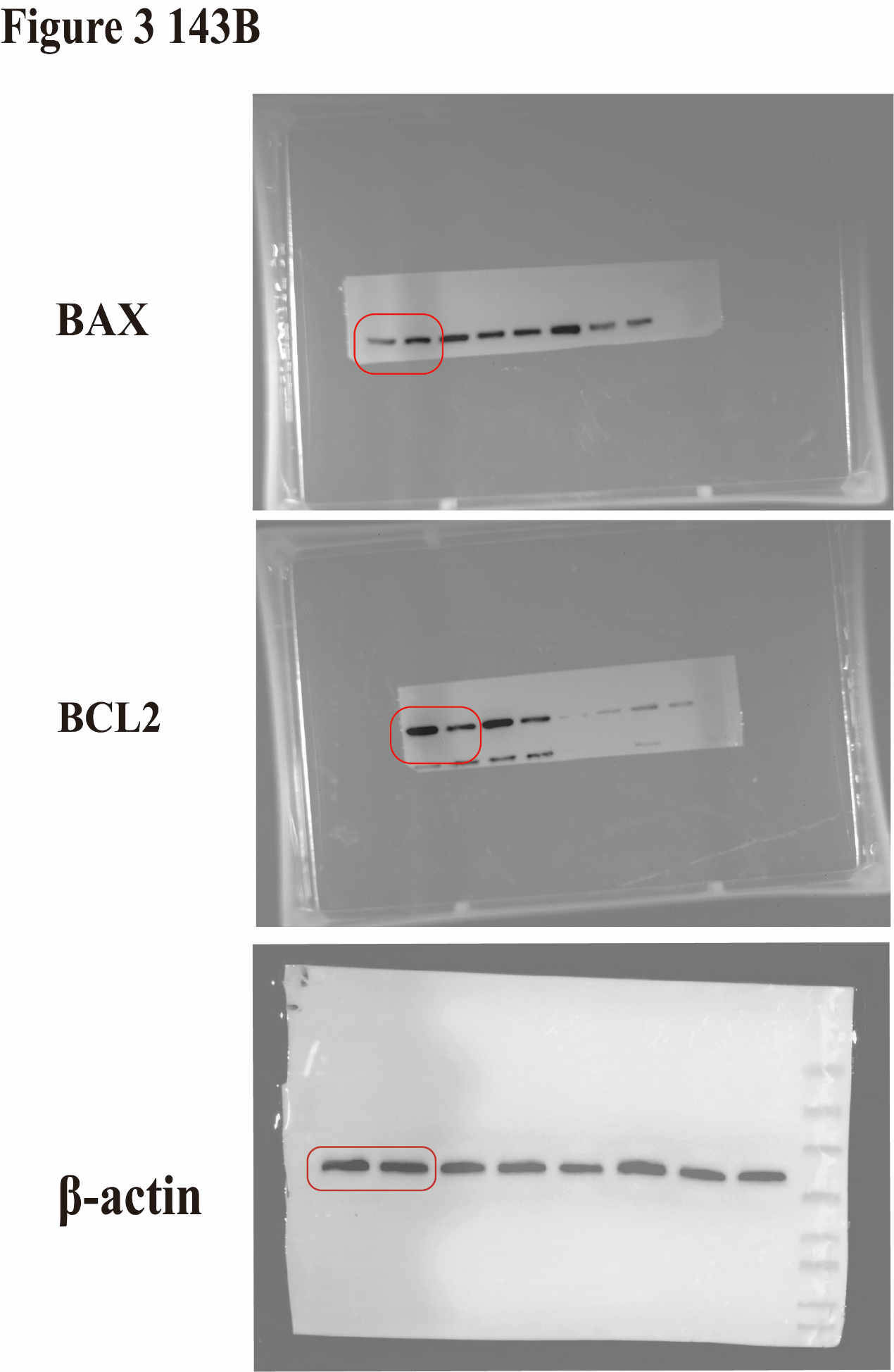


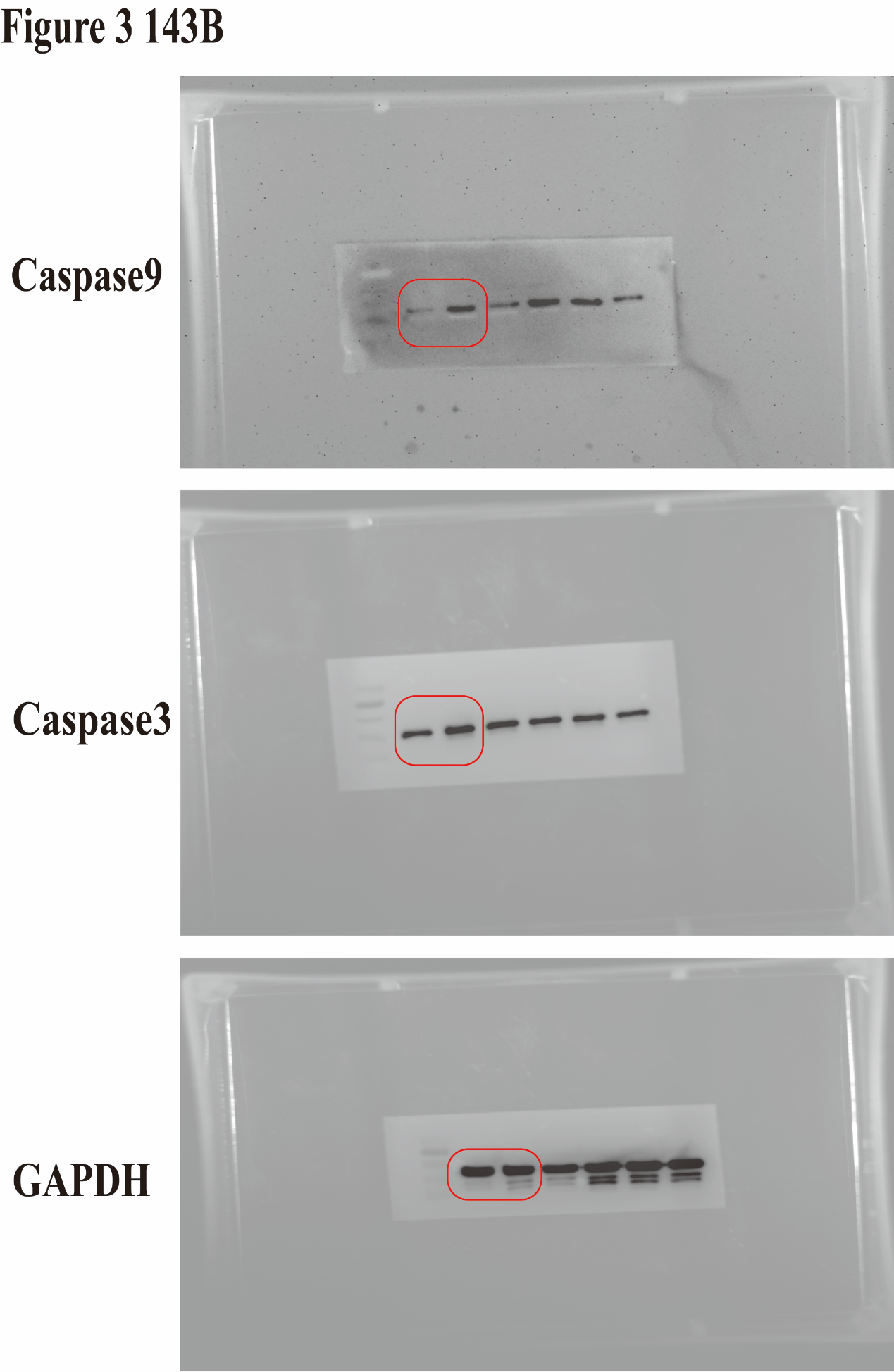


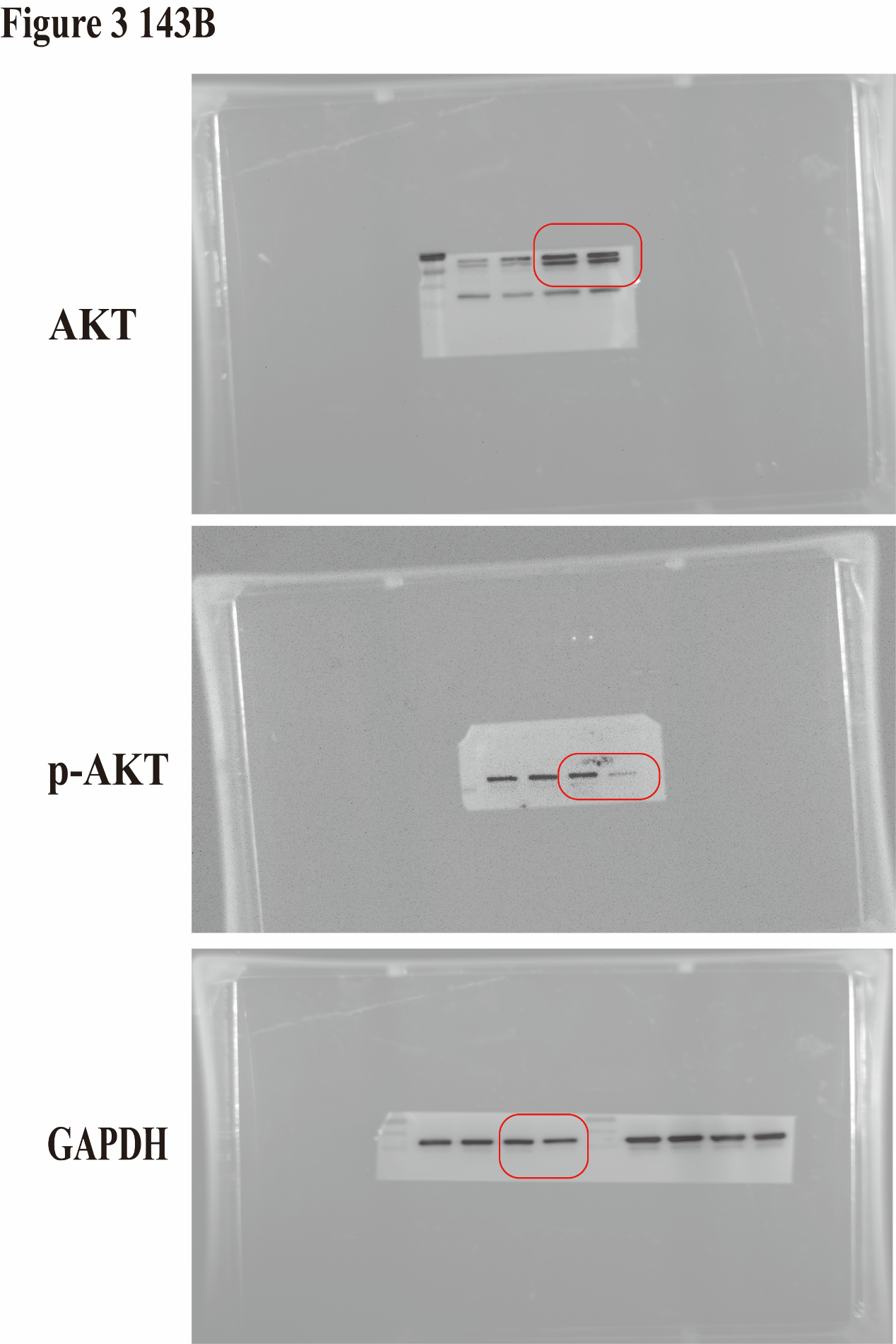


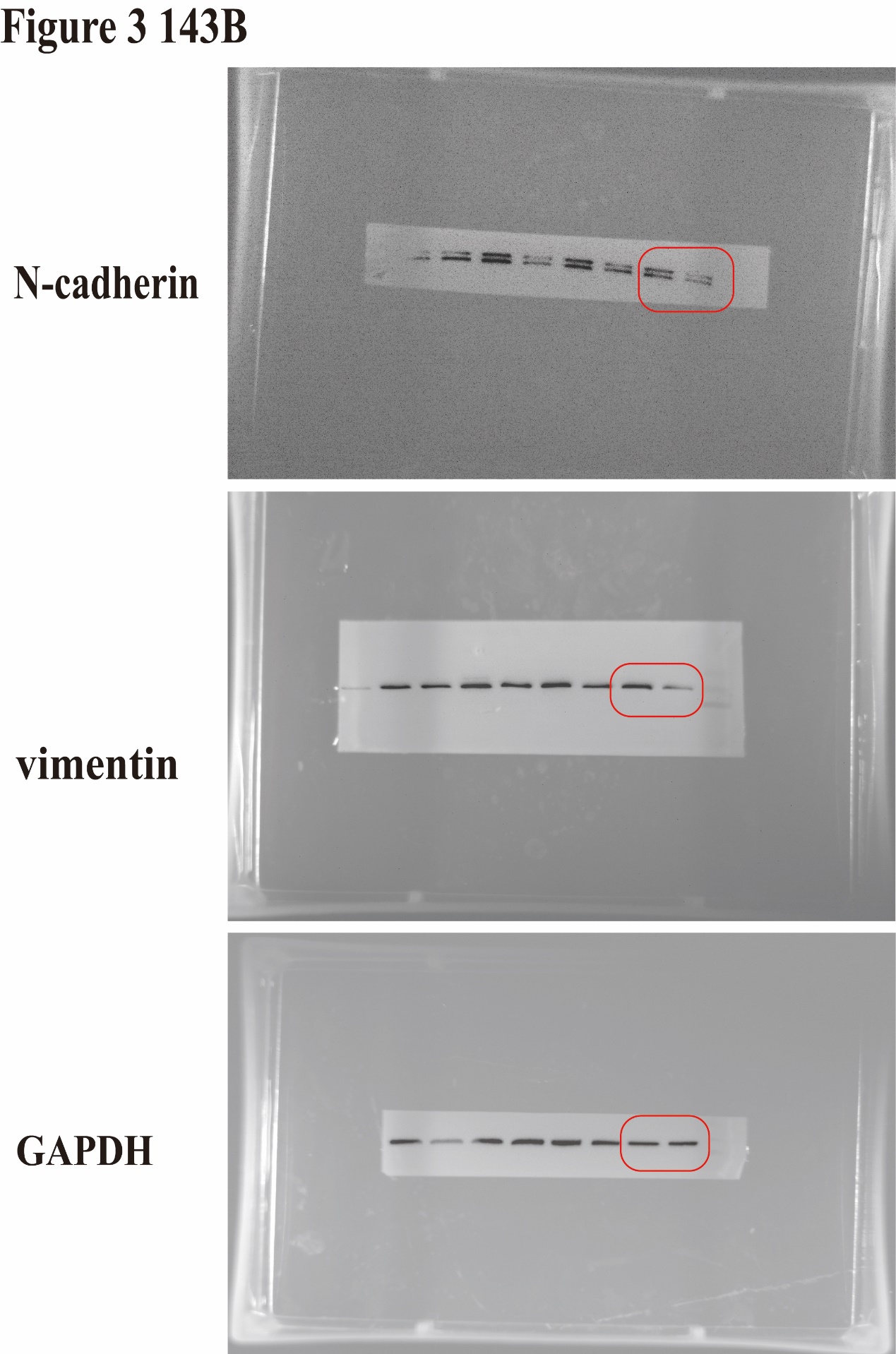


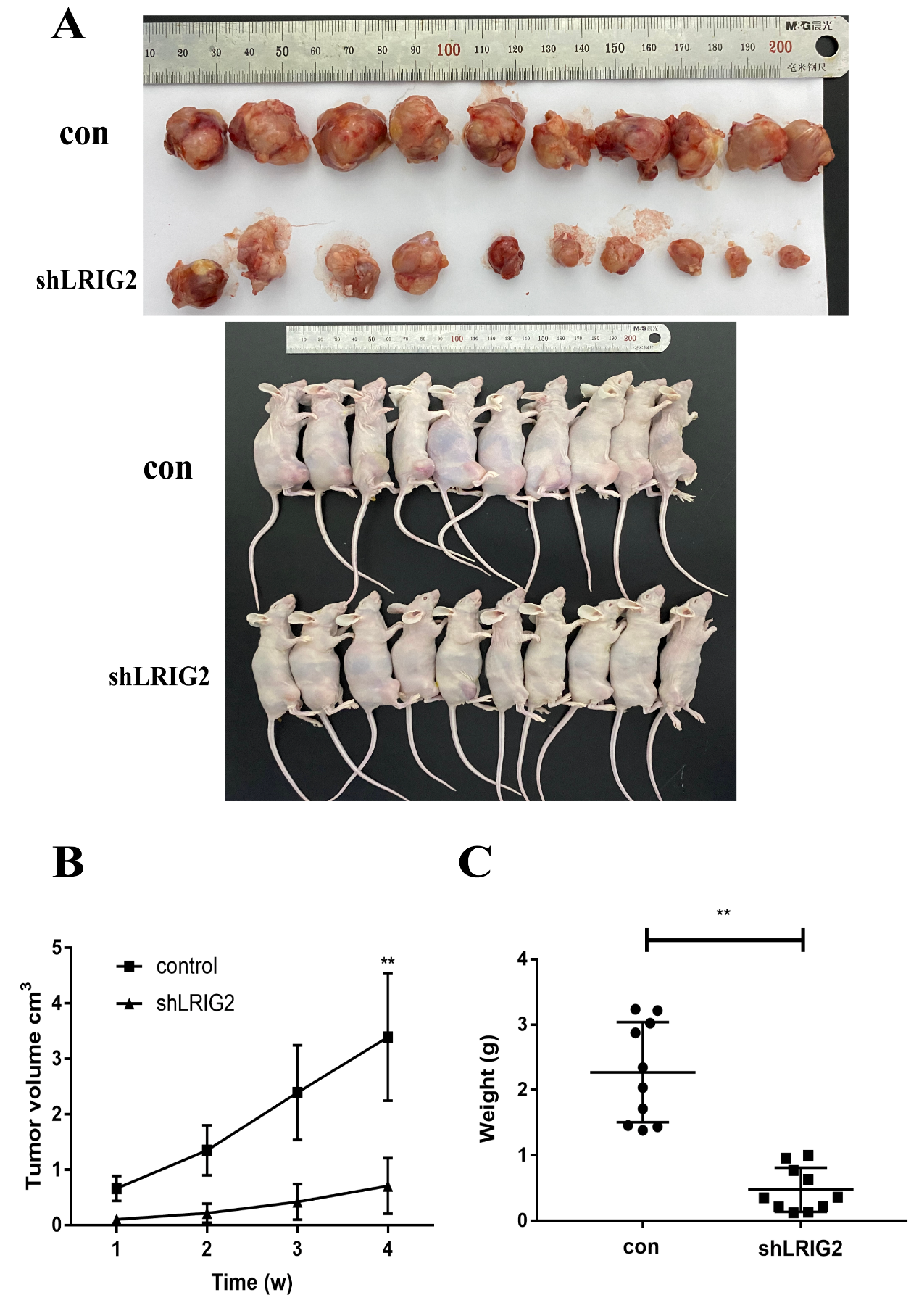


**Figure 4.** Silencing LRIG2 inhibits the growth of osteosarcoma spontaneous metastasis xenograft models. (A) Orthotopic osteosarcoma xenograft tumor models were established, the nude mice were euthanized after 4 weeks, orthotopic tumors dissected to obtain samples. (B) Tumor sizes were measured weekly and calculated using the following formula: V= (Length×Width^2/2). (C) Tumors were dissected and weighted.


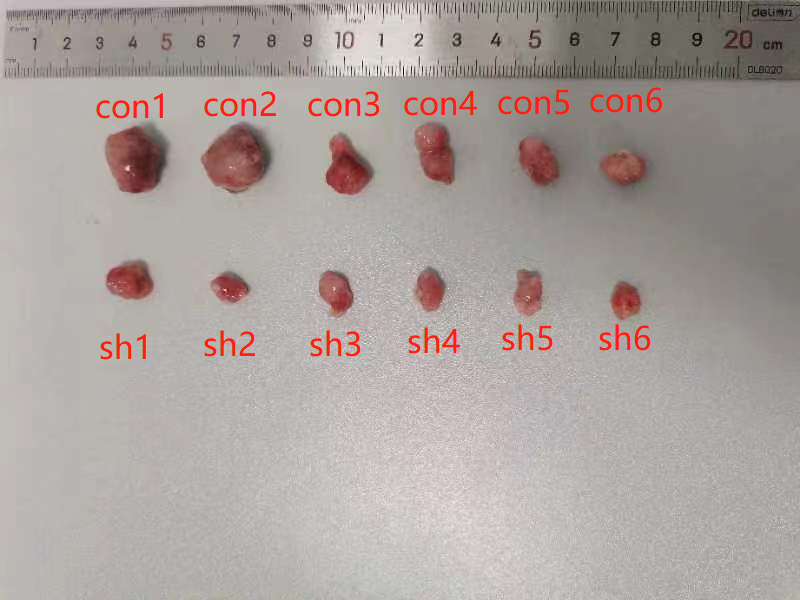


**
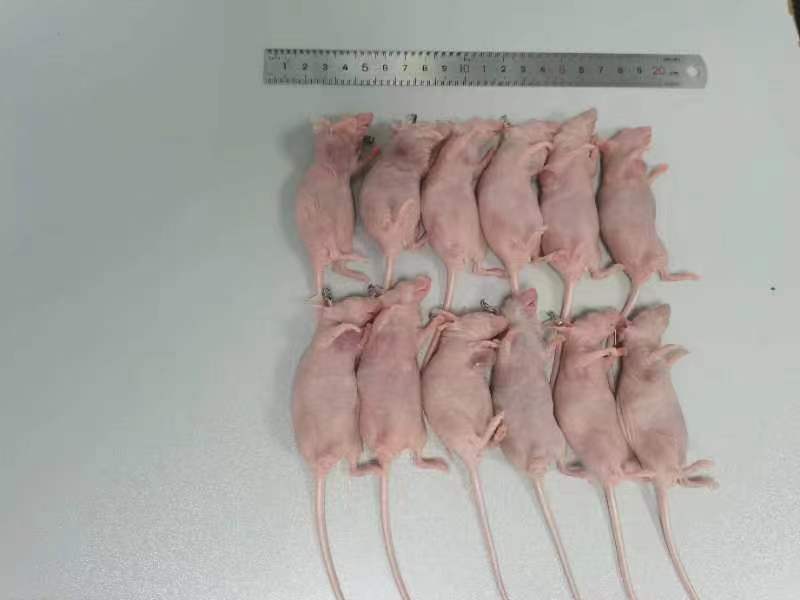
**


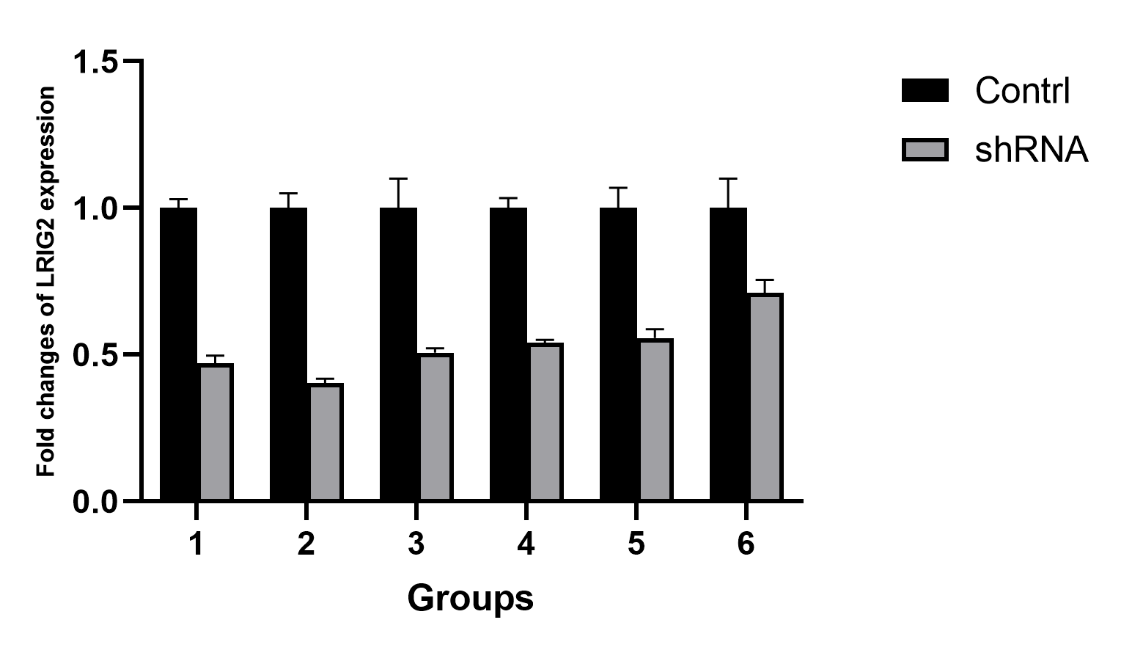


**Figure 5.** Silencing LRIG2 inhibits the growth of osteosarcoma xenograft tumor. LRIG2 stably transfected gene silencing were inoculated subcutaneously into the nude mice. The nude mice were euthanized after 5 weeks, subcutaneous tumors dissected to obtain samples (6 for control group and 6 for LRIG2- knockdown group). The relative mRNA expression levels of control group and LRIG2-knockdown group were detected by qRT-PCR.
